# Fatigue in Systemic Lupus Erythematosus and Rheumatoid Arthritis: A Comparison of Mechanisms, Measures and Management

**DOI:** 10.3390/jcm10163566

**Published:** 2021-08-13

**Authors:** Mrinalini Dey, Ioannis Parodis, Elena Nikiphorou

**Affiliations:** 1 Institute of Life Course and Medical Sciences, University of Liverpool, Brownlow Hill, Liverpool L69 3BX, UK; 2 Department of Rheumatology, Aintree Hospital, Liverpool University Hospitals NHS Foundation Trust, Lower Lane L9 7AL, UK; 3 Division of Rheumatology, Department of Medicine Solna, Karolinska Institutet and Karolinska University Hospital, 171 76 Stockholm, Sweden; ioannis.parodis@ki.se; 4 Department of Rheumatology, Faculty of Medicine and Health, Örebro University, 701 85 Örebro, Sweden; 5 Rheumatology Department, King’s College Hospital, London SE5 9RJ, UK; elena.nikiphorou@kcl.ac.uk; 6 Centre for Rheumatic Diseases, King’s College London, London SE5 9RJ, UK

**Keywords:** systemic lupus erythematosus, rheumatoid arthritis, fatigue, quality of life, pain, psychosocial, disease activity

## Abstract

Fatigue is a common constitutional feature of systemic lupus erythematosus (SLE) and rheumatoid arthritis (RA). While the two diseases share a common mechanism of autoimmunity, they differ in their clinical manifestations and treatment. Fatigue is one of the most commonly reported symptoms in both groups, associated with pain, depression and anxiety, and affecting function, work and quality of life. Fatigue is not easy to assess or conceptualise. It can be linked to disease activity, although it is not always, and is challenging to treat. Several measures have been trialled in RA and SLE; however, none have been adopted into mainstream practice. Despite being a common symptom, fatigue remains poorly managed in both RA and SLE—more so in the latter, where there have been relatively fewer studies. Additionally, comorbidities contribute to fatigue, further complicating its management. Pain, depression and anxiety also need to be addressed, not as separate entities, but together with fatigue in a holistic manner. Here, we describe the similarities and differences between fatigue in patients with RA and SLE, discuss concepts and practices applicable to both conditions and identify areas for further research. Through this review, we aim to highlight the importance of the holistic management of fatigue in SLE.

## 1. Introduction

Fatigue is a subjective symptom of malaise and aversion to activity, comprising both physical and mental aspects [1]. It is often poorly defined in clinical practice and may be reported by patients as “fatigue”, “tiredness”, “lethargy” or “exhaustion”, as well as other descriptors for a lack of energy [1]. It is not easy to assess or conceptualise. Fatigue is one of the most frequent presentations in primary care, affecting up to 20% of the general population, and is twice as common in women than in men [2,3]. In the case of chronic disease, up to 50% of people experience fatigue as part of their condition.

The prevalence of fatigue sharply increases when considering rheumatic diseases. For decades, it has been known that fatigue is one of the most commonly reported symptoms, affecting almost all patients [4]. Rheumatoid arthritis (RA) and systemic lupus erythematosus (SLE) are two rheumatic diseases where fatigue features strongly as one of the predominant symptoms, beyond the articular and connective tissue disease features. Significant fatigue is reported by two-thirds of patients with SLE, and severe fatigue is reported by one-third of these patients, as defined by the Fatigue Scale for Motor and Cognitive Functions scale (FSMC)—a self-administered questionnaire initially developed for patients with multiple sclerosis [5,6,7,8]. Up to 75% of RA patients experience persistently high or worsening levels of fatigue [9]. While the two diseases share a common mechanism of autoimmunity, they differ in their underlying immunopathology, treatment and resulting clinical manifestations, such as organ involvement. Despite these differences, fatigue is one of the most commonly reported symptoms in both patient groups, and is associated with symptoms of pain, depression and anxiety, while impacting function, work and overall quality of life [7,10,11].

Fatigue may be linked to disease activity, although it is not always, and can be challenging to treat. Several scores and measures of fatigue have been trialled in RA and SLE, with variable success, and none have been adopted into mainstream clinical practice. Despite being one of the most troubling symptoms reported by patients, fatigue remains poorly managed in both RA and SLE. It is important to rule out causes of fatigue not related to the primary rheumatological diagnosis, and aim for optimal disease control. Additionally, the comorbidity profile of patients with SLE and RA differs greatly, e.g., renal disease is more common in SLE [12,13]. Comorbidities may be directly or indirectly related to the primary diagnosis and are likely to contribute to the burden of fatigue in both patient groups, further complicating its management. Related factors such as pain, depression and anxiety also need to be addressed, not as separate entities, but together with fatigue to ensure a holistic approach to management.

In this review, we describe the similarities and differences between fatigue and its associations in patients with RA and SLE, discuss the concepts and practices that may be applied in the two conditions, compare and contrast the measures of fatigue and identify areas for further research on fatigue in SLE. Through this review, we wish to highlight the importance of the holistic management of fatigue in SLE, addressing all possible causes, as a symptom that is intertwined with the other aspects of the disease.

## 2. Recognising Fatigue as a Clinical Outcome

Despite its long being recognised as a key symptom in patients with RA and SLE, healthcare professionals and researchers have only recently started to appreciate the clinical relevance of fatigue, its impact on patients (and all aspects of their lives) and the need for appropriate assessment and suitable, discriminative outcome measures. This was largely prompted by a Patient Perspective Workshop at an OMERACT meeting in 2002, with a subsequent formal recommendation in 2006, highlighting the importance of recognising fatigue as a core outcome measure amongst patients with RA [14,15,16]. Both qualitative and quantitative studies have, subsequently, demonstrated the high prevalence of fatigue amongst patients with RA, encompassing physical, cognitive, social and emotional fatigue, indicating the need to tailor fatigue management to the individual situation of the patient [17,18].

While fatigue is also recognised as highly prevalent amongst patients with SLE, quantifying this and incorporating it into the holistic assessment of the patient has proved challenging, not only due to the multidimensional impacts of fatigue, but also because of the complex aetiology of this multi-system disease. The assessment of fatigue becomes particularly challenging in patients without active disease. The extent to which disease activity plays a role remains uncertain, and there is a clear (albeit complex) association with depression and anxiety, as well as related symptoms of chronic pain and fibromyalgia, and disordered sleep [19].

## 3. The Role of Disease Activity

**Figure 1:**
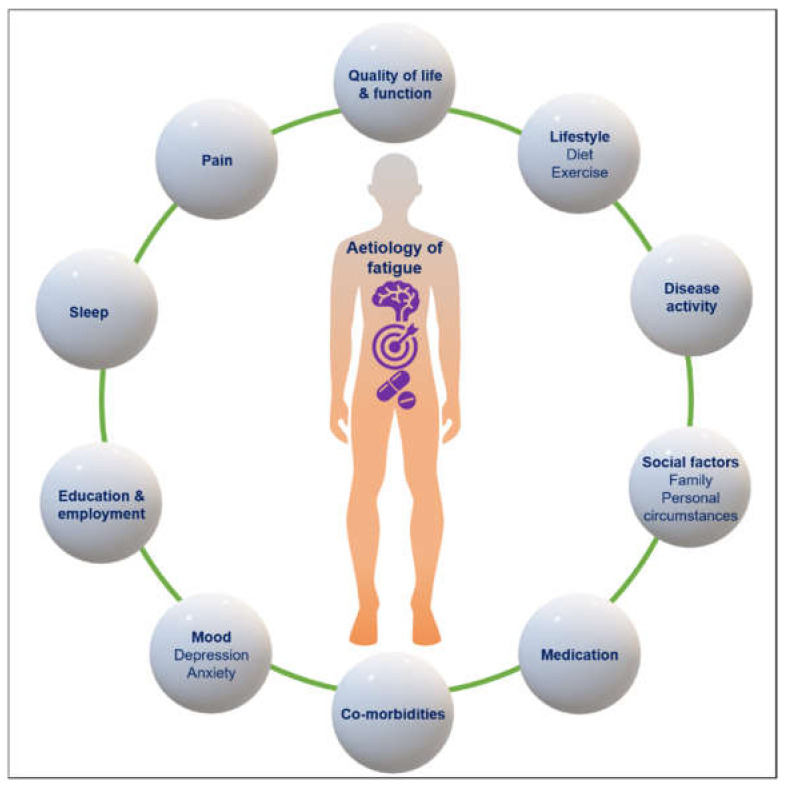
Factors associated with fatigue in rheumatoid arthritis and systemic lupus erythematosus. Fatigue is a complex, multi-factorial symptom. Multiple aspects of a patient’s biological, social and psychological circumstances contribute; this is not an exhaustive list. Examples of broad contributory factors, such as mood, lifestyle and social factors, are provided below each heading.

The aetiology of fatigue and its association with disease activity remains contentious in both RA and SLE (Figure 1). It has been suggested that systemic inflammation may contribute to fatigue. In the case of RA, the primary manifestations of pain, joint problems and functional limitations (which contribute to disease activity scores) may also play a role [20]. Fatigue is a major contributor to patients’ global assessment, and it has been suggested, based on trial data, that it is a separate aspect of disease, which may be explored as a treatment target in its own right, separate from disease activity [21,22].

Similar findings have been demonstrated in patients with SLE. The FATILUP study was a large observational study assessing the determinants of fatigue in 570 patients with SLE [7]. This study found a significant but limited association between SLE disease activity and fatigue, with an accompanying systematic literature review concluding that there is no major role for disease activity, albeit with some studies reporting a link with neurological involvement [7]. It is notable that, in the FATILUP cohort, arthritis and ulcers showed the strongest associations with fatigue, which, again, suggests a role for pain in its aetiology.

It has been hypothesised that inflammatory molecules (often raised in active RA), such as tumour necrosis factor (TNF), interleukin-6 (IL-6) and C-reactive protein (CRP), may contribute to fatigue symptoms. However, the evidence is inconsistent [20]. Early studies in mouse models demonstrated that high levels of IL-1 and IL-6 induce fatigue and hypersomnia, which can be resolved with the administration of anti-inflammatory drugs [23,24,25]. However, in human patients, the erythrocyte sedimentation rate (ESR) only poorly correlates with fatigue, with mixed evidence for CRP [26,27,28]. While these components of disease activity have a significant association with fatigue, one systematic review found this association to be mainly driven by pain [27]. Looking more widely at the other components of disease activity scores, fatigue was only weakly associated with the swollen joint count in one cohort, while a large longitudinal study found a significant, but small, association with both tender and swollen joint counts that did not resolve after improved treatment strategies [27,28]. Furthermore, when considering the evidence of inflammation on joint imaging, such as MRI, patients with greater levels of MRI-detected inflammation are not necessarily those with more severe fatigue, suggesting that fatigue is, at least in part, a separate entity from inflammation [29].

The evidence base is even more sparse when we consider SLE. The FATILUP study found some components of the SLE disease activity score (SLEDAI), arthritis and oral ulcers, to be associated with fatigue, although they are likely to be confounded by pain [7]. A large longitudinal cohort study found no association with disease activity and fatigue, and this has since been repeated in other cohorts [30,31]. However, other studies have shown decreasing fatigue levels following therapy, including signals of a protective role for belimumab against severe fatigue [32,33,34,35]. It is important to consider the role of fibromyalgia in these patients, as it is present in up to 25%, and has a strong association with fatigue, as well as related symptoms of depression, anxiety and poor sleep [5,31]. A recent study examining fatigue trajectories in a cohort of SLE patients found no association with disease activity, but identified higher levels of fatigue in those with fibromyalgia. This study also noted an association between higher glucocorticoid use and fatigue, suggesting a role for the side effects of medication, particularly glucocorticoids [36]. Crucially, while many studies have examined the relationship between inflammatory molecules and organ involvement, such as lupus nephritis, very few studies have been performed examining fatigue—in stark contrast to RA.

## 4. Mechanisms of Fatigue and Association with Other Symptoms

In both RA and SLE, fatigue may be correlated with other organ involvement, such as renal, cardiac, respiratory and neurological diseases. This is particularly relevant to SLE. However, something which is important and common to fatigue in both of these conditions is mental health outcomes. 

**Figure 2:**
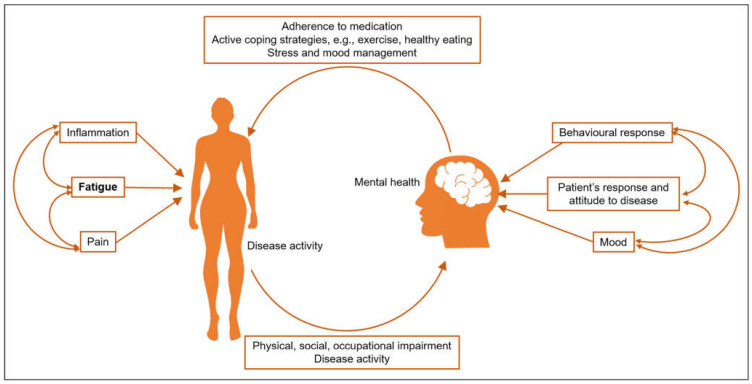
The relationship between fatigue and mental health in rheumatic diseases [39].

Fatigue, poor sleep and depression are closely intertwined, with a recent study in RA patients demonstrating greater severity and frequency of depressive symptoms with poorer sleep [37]. This has wider psychosocial impacts, such as decreased quality of life, work participation and physical functioning [20,38,39] (Figure 2). These diverse impacts on mental health and quality of life mean the experience of fatigue for each individual patient is unique and requires a personalised approach to management, something which has been highlighted through qualitative work in this area [18]. Mental health outcomes are also strongly associated with fatigue in SLE, affecting approximately 13% of patients with SLE, and are attributed to the disease in 40% of cases [5]. A recent cross-sectional study in the California Lupus Epidemiology cohort found that a quarter of the 326 patients met the criteria for major depression, with these individuals being more likely to have greater levels of fatigue and sleep impairment, negative psychosocial impacts of illness, decreased satisfaction in discretionary social activities and decreased satisfaction in social roles [40]. The FATILUP study found a similarly high prevalence of depression and anxiety. In total, 44.4% of patients with fatigue reported one or both of these conditions, rising to almost 60% in those with severe fatigue, with odds of between four and seven for the association between fatigue or severe fatigue and depression or anxiety [7]. Stress, pain and depression are the largest contributors to fatigue in patients with SLE, indicating a need for specialist assessment and management [5,31]. It is clear that depression and anxiety are highly prevalent in people with either RA or SLE and that they are closely associated with fatigue. However, the management of these symptoms within the context of the patient’s disease and wider health and well-being remains poor, impacted by factors such as inadequate access to services (such as psychology) and a continued need for greater awareness amongst clinicians [41].

The similarities between the aetiology and manifestation of fatigue in RA and SLE become less clear when considering multi-organ involvement. Sjögren’s syndrome is prevalent in both conditions, affecting 15–30% of both patient groups, and has been associated with fatigue in multiple studies, independent of disease activity or pain conditions such as fibromyalgia [42,43,44,45]. 

Importantly, SLE is characterised by multi-organ involvement. The skin, cardiovascular system and central nervous system are frequently affected, and over half of patients with SLE develop lupus nephritis during their disease course [46]. Most organ involvement in SLE contributes, to some extent, to a patient’s overall fatigue, partially explaining its high prevalence. The most frequently self-reported symptom in patients with kidney disease is fatigue, affecting up to two-thirds of non-dialysis patients and being associated with factors such as comorbidity burden, anaemia and the use of anti-depressants [47,48]. The latter may reflect the mental health burden in chronic disease manifesting as fatigue. Anaemia is one plausible explanation for the aetiology of fatigue in these patients, and is often present, even in the absence of kidney disease, in SLE. Anaemia is also present in patients with cardiac failure, a manifestation of SLE (a common comorbidity in those with RA), although few studies have sought to quantify the impact of cardiac failure in either condition [20]. Cardiovascular disease, including ischaemic heart disease, correlates with RA disease activity more than any other comorbidity, including a significant association with patient-reported fatigue [49]. Similar studies on patients with SLE are lacking, signifying a critical gap in the evidence, given the prevalence of heart disease in these patients.

Neurological involvement is seen in both SLE and RA, manifesting in different ways. Common symptoms in RA include neuropathy secondary to atlantoaxial subluxation, mononeuritis multiplex and peripheral neuropathy [50]. Central nervous system involvement is rare but can present as, for example, cerebral rheumatoid vasculitis [51]. Crucially, few studies have considered the effects of these neurological manifestations on fatigue prevalence in RA. The topic remains relatively understudied in SLE, though some evidence is beginning to emerge for certain types of neurological involvement [52]. The chronic inflammatory state in SLE, mediated by pro-inflammatory cytokines, promotes oxidative and nitrosative stress. This results in the production of damage-associated molecular patterns and the engagement of toll-like receptors, which then manifest as fatigue. Interferon-α (IFNα) is involved in SLE disease pathogenesis and is associated, in a dose-dependent manner, with neuropsychological symptoms, including fatigue, as well as depression and seizures [53,54]. Specifically, increased white matter hyperintensities have been observed in patients with SLE, associated with increased fatigue, similar to patients with multiple sclerosis [55,56].

Other related clinical states, such as vitamin D deficiency, as well as the use of certain medications, also contribute to fatigue, particularly the use of corticosteroids, which are commonly used in both SLE and RA patients [57,58]. The side effects of corticosteroids, particularly insomnia and weight gain, are both independently associated with fatigue. In an individual with active SLE or RA, fatigue is, therefore, often compounded by the use of corticosteroids. Lastly, fatigue in SLE has been shown to be associated with overweight and obesity in a body mass index (BMI)-level-dependent manner [59].

## 5. Measures of Fatigue

Fatigue in both RA and SLE has a complex aetiology and presentation, and is highly subjective. This makes it exceptionally difficult to quantify and compare, even between patients with the same disease. Patient-reported outcomes are crucial. Despite being a required outcome for clinical trials in RA, as mandated by The American College of Rheumatology and European Alliance of Associations for Rheumatology, there are no formally recommended fatigue scores for clinical use. In SLE, no such mandate exists; however, due to the increasing awareness of fatigue as a major symptom in these patients, much work has been done to develop such a tool [5,20,60].

Multiple fatigue scales exist for use in both SLE and RA. Recent reviews have identified 16 such tools in SLE and 23 in RA, with some overlap between the two diseases [5,20,61,62]. Many scales have been created for the purposes of a given study and are, therefore, not necessarily validated (the most commonly used measures are summarised in Table 1). Since these reviews, the Patient-Reported Outcomes Measurement Information System (PROMIS) has been developed for use in RA, although work to identify clinically meaningful results with regard to the magnitude of change in symptoms is ongoing [63,64,65].

In addition to formally recognising the burden of fatigue in patients, a validated measure enables the clinician to monitor symptom burden and the response to treatment over time. The commonly used scores across both SLE and RA include Visual Analogue Scales (VAS), SF-36 Vitality subscale score and the Functional Assessment of Chronic Illness Therapy–Fatigue (FACIT-Fatigue) score. Others, such as the Bristol Rheumatoid Arthritis Fatigue-Multidimensional Questionnaire (BRAF-MDQ) and Fatigue Severity Scale (FSS) have been favoured for either RA or SLE, respectively [5,66]. The BRAF-MDQ was designed in collaboration with patients, capturing the multiple facets of fatigue, including emotional, psychological and cognitive burden, as well as allowing clinicians to evaluate the impacts of fatigue on the patient’s function. Going forward, it is essential to ensure that patients are involved in the development of tools for assessing fatigue, given the variation in experience and impact across patients.

Rather than “reinventing the wheel”, it is also essential to look across the rheumatic diseases to assess the performance of each tool. It may be argued that the existence of multiple fatigue scores across SLE and RA alone demonstrates the need for one common measure across the rheumatic diseases, given the prevalence of fatigue in these patients. However, as discussed, there are subtle differences in the aetiology and characteristics of fatigue across patient groups. It may, therefore, be some time before a consensus is reached on this front.

## 6. Management of Fatigue

There is no single treatment for fatigue in either RA or SLE. It may be argued that better disease control may abate symptoms; however, as discussed, the aetiology is complex and remission does not equate to the absence of fatigue [67]. Nonetheless, optimal disease control is essential to reduce the inflammation-driven component of fatigue. A 2016 Cochrane review concluded that biologic DMARDs elicit a moderate reduction in fatigue when measured with FACIT-F and the SF-36 vitality subscale [68,69]. Specifically, the use of TNF inhibitors was found to reduce fatigue by 6.3 units on FACIT-F, or 7.5 units on SF-36, compared to the controls, based on 19 studies with 8946 patients. The use of other biologics produced an even greater effect, leading to a reduction in FACIT-F of 6.9 units or 8.19 on SF-36, compared to the controls, based on eleven studies with 5682 patients. Since the publication of this Cochrane review, Janus kinase (JAK) inhibitors have been licensed for use in RA treatment. Tofacitinib and baricitinib have been shown to significantly reduce fatigue in patients in clinical trials. Specifically, there were significant reductions in the FACIT-F fatigue scores, and improvements in work productivity, pain and function, compared to placebo and methotrexate, particularly in inadequate responders [68,70,71,72,73]. Data from all four of the major RA studies with baricitinib, at both 2 mg and 4 mg dosing, showed that 63–75% of participants had improvements in FACIT-F scores after 12 weeks, compared to 48–65% of patients in the control groups [71,72,73]. The use of JAK inhibitors and most biologics are limited to RA, highlighting an area for further research in SLE. It is also important to note that not all patients will respond to treatment in the same way, particularly with regards to a multi-facetted symptom such as fatigue.

Treatment-related fatigue should also be addressed, and is not limited to the use of corticosteroids. Fatigue has been found to be one of the main causes of methotrexate non-adherence in quantitative studies, although evidence from qualitative studies demonstrates a variation in patients’ experiences of fatigue when taking this drug [74,75].

**Figure 3:**
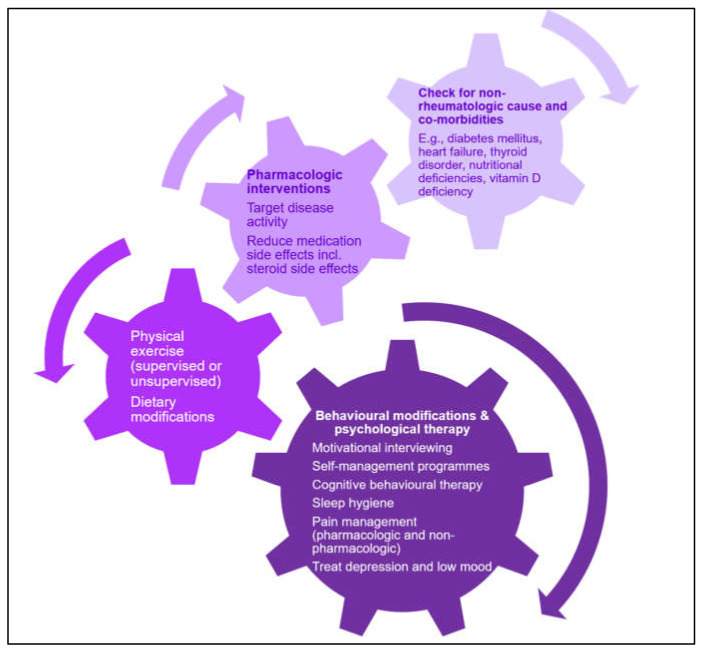
Common themes in the management of fatigue in rheumatoid arthritis and systemic lupus erythematosus [5,62].

The management of associated symptoms of depression, anxiety and pain may be helpful. Given the overlap in characteristics between fatigue in RA and SLE in this regard, this is an area where similar management strategies may be effective for both (Figure 3). The empowerment of patients to undertake the self-management of their fatigue is crucial, given the disparate characteristics of this symptom between patients. The recent EULAR guidance on the self-management of symptoms in inflammatory arthritis describes the benefits of this for both patients and clinicians, including a more holistic patient-centred approach, ultimately leading to better outcomes [76]. Approaches include psychosocial and physical approaches, with appropriate input, as required from health professionals [76,77,78]. Group cognitive behavioural therapy has also proven effective in reducing the burden, impact and severity of fatigue in RA. Physical exercise and psychological interventions also have proven benefits for fatigue in SLE, as well as depression and quality of life, demonstrating the similarities in effective strategies between the two conditions [79]. More simple measures, such as encouraging smoking cessation and a healthy diet, are likely to potentiate the effects of such self-management strategies—particularly the former, which adversely affects disease activity and response to treatment in both RA and SLE [33,80,81,82].

In severe or unremitting cases of fatigue, psychological assessment should be sought, with the appropriate behavioural or psychological interventions. Unremitting and debilitating fatigue for more than six months warrants further assessment for other possible co-existing causes, such as chronic fatigue syndrome [83]. The role of antidepressants in fatigue in SLE requires particularly careful thought, due to potential interactions with hydroxychloroquine and prolonged QT [5]. They may be more suitable in the setting of RA, provided the patient is not also taking hydroxychloroquine or a similar interacting medication, although evidence on this is limited. 

Ultimately, across both conditions, due to the multifactorial aetiology of fatigue and its impact on multiple facets of patients’ lives, a holistic approach to its management is vital, tailored to the patient’s individual needs and circumstances.

## 7. Conclusions and Future Research Considerations

Fatigue is one of the most prevalent and debilitating symptoms in patients with rheumatic diseases. Although differing in pathology and symptomology, it is clear that there is much overlap in the nature, associated features (pain, low mood, poor sleep, por function) and proposed management of fatigue in both of these conditions. However, it is also appropriate to appreciate the differences, such as the impact of different associated organ involvement, treatments and disease activity in determining the aetiology of and, therefore, the finer ways of managing fatigue.

Multiple measures and scales have been trialled to assess fatigue in both RA and SLE. Despite a universal recognition of the importance of appropriately measuring (and addressing) fatigue, a single standardised measure, even within a single condition, is lacking. Due to the similarities in the various aspects of fatigue across both RA and SLE, a single tool of measure across such diseases may encourage clinicians to measure and place greater focus on this symptom when assessing and managing patients. Research in this area would also be greatly aided by a single tool. However, the aetiology and more detailed characteristics of fatigue in RA and SLE are sufficiently different to warrant disease-specific measures, for example, accounting for co-existing organ involvement, neurological function, disease activity measures and treatment.

Ultimately, the improved management of fatigue in patients with rheumatic diseases will lead to better overall well-being and encourage a holistic approach to patient care. Self-management strategies are crucial to relieving fatigue and related symptoms of pain, low mood and disturbed sleep, and have an overall impact on general health, function and disease activity. The assessment of fatigue in clinical practice remains infrequent. More robust and simple measures, and heightened awareness amongst clinicians and patients of the multifactorial nature of fatigue, is likely to improve its management.

## Figures and Tables

**Table 1:** Summary of the most commonly used measures for assessment of fatigue used in systemic lupus erythematosus (SLE) and rheumatoid arthritis (RA) [5,11].

SLE and RA		RA Only	
Fatigue Severity Scale (FSS)	9-item scale covering psychosocial and cognitive aspects of fatigue. Originally developed for use in multiple sclerosis and SLE.	Bristol Rheumatoid Arthritis Fatigue Multi-Dimensional Questionnaire	20-item scale assessing the experience and impact fatigue, giving an overall score comprising 4 subscale scores (physical fatigue, living with fatigue, cognitive fatigue, emotional fatigue).
Multi-dimensional Fatigue Inventory (MFI)	20-item scale comprising 5 domains: general fatigue, physical fatigue, mental fatigue, reduced motivation, reduced activity. Significant fatigue is defined depending on age and gender.	Bristol Rheumatoid Arthritis Fatigue Numeric Rating scales	3 scales, scored 0–10: severity (no fatigue–totally exhausted), effect (no effect–a great deal of effect), coping (not at all well–very well).
Visual analogue scale to evaluate fatigue severity (VAS-F)	18-item scale based on subjective experience of fatigue, using fatigue and energy subscales	Checklist of Individual Strength (CIS20)	20-item scale giving overall score comprising 4 sub-scores (subjective fatigue, concentration, motivation, physical activity levels).
Functional Assessment of Chronic Illness Therapy–Fatigue (FACIT-Fatigue)	13-item questionnaire on self-reported aspects of physical, mental and functional fatigue, and effect of these on daily living.	Fatigue Severity Inventory	11-item scale comprising 2 scores: 6 items rating average fatigue in past week, on days with most and least fatigue, number of days with fatigue, duration of fatigue each day and current fatigue levels; 5-item fatigue interference scale.
		Multidimensional Assessment of Fatigue (MAF)	15-item scale comprising 4 aspects of fatigue (severity, distress, ability to undertake activities of daily living, frequency and change during previous week).
		Profile of Mood States (POMS)	7-item scale, focussing mainly on mood plus cognitive components and overwhelming fatigue.
		SF-36 (36-Item Short Form Survey)	4-item score, 2 on energy and 2 on fatigue.
		Patient-Reported Outcomes Measurement Information System (PROMIS) Fatigue scales	Scale ranging from subjective feelings of tiredness to overwhelming exhaustion impacting activities of daily living.

All scores have been used in the assessment of patients with RA, with four also being used in patients with SLE. No single universally agreed score for the assessment of fatigue within either diagnosis or across SLE and RA exists.
